# A review of the effectiveness of telemedicine in glycemic control in diabetes mellitus patients

**DOI:** 10.1097/MD.0000000000032028

**Published:** 2022-12-02

**Authors:** Clara Fernanda Kusuma, Levina Aristawidya, Chairani Putri Susanti, Angga Prawira Kautsar

**Affiliations:** a Undergraduate of Pharmacy, Faculty of Pharmacy, Universitas Padjadjaran, Sumedang, West Java, Indonesia; b Center of Excellence in Higher Education for Pharmaceutical Care Innovation, Universitas Padjadjaran, Sumedang, West Java, Indonesia; c Department of Pharmaceutics and Technology of Pharmacy, Faculty of Pharmacy, Universitas Padjadjaran, Sumedang, West Java, Indonesia; d Unit of Global Health, Department of Health Sciences, University of Groningen (RUG)/University Medical Center Groningen (UMCG), Groningen, The Netherlands.

**Keywords:** blood glucose, glycemic control, counseling, diabetes mellitus, RCT, telemedicine

## Abstract

This review aimed to evaluate the effectiveness of telemedicine as an intervention for patients with diabetes mellitus, considering blood glucose levels as the primary outcome. A comprehensive literature search was performed using the PubMed, Embase, Web of Science, and EBSCO databases. This narrative review covered randomized controlled trials published in English. The process of selecting studies adhered to the preferred reporting items for systematic reviews and meta-analyses guidelines. Nine studies were selected, and their data were analyzed and summarized. Five of the nine reviewed studies found that telemedicine counseling was effective in decreasing glycated hemoglobin A (HbA1c) levels in the blood. Due to methodological limitations, one study could not report HbA1c results, and two studies reported that telemedicine counseling did not lead to any significant changes in HbA1c levels. One study found that while HbA1c levels did not show a marked decrease, patients’ treatment adherence and quality of life improved when telemedicine was combined with health counseling. Moreover, six studies found that telemedicine counseling was more effective than traditional counseling regarding secondary outcomes. The overall findings of this review suggest that telemedicine counseling is more effective than conventional counseling in achieving decreased blood glucose levels in patients with diabetes mellitus while increasing their treatment adherence and improving their quality of life.

## 1. Introduction

The global burden of diabetes mellitus (DM) and its prevalence continually increase. The global incidence of DM escalated by 102.9% between 1990 and 2017, rising from 11.3 to 22.9 million, with type 2 DM (T2DM) accounting for most cases. This trend is likely to continue to do so until 2025. In the absence of effective interventions, the increase is projected to rise to 570.9 million prevalence, 1.59 million mortality, and 79.3 million disability-adjusted life years.^[1]^ According to the International Diabetes Federation Diabetes Atlas (2021), 537 million adults aged 20 to 79 currently have diabetes. This number is estimated to increase by 46% between 2021 and 2045. Projections of the prevalence of adults with DM worldwide in 2030 and 2045 are 643 and 783 million, respectively.^[2]^

High glucose levels in patients with DM are caused by a lack of insulin production or conditions, thereby impeding the transfer of glucose in the blood into the cells. T2DM is associated with pancreatic β-cell dysfunction coupled with some degree of insulin resistance. Dysfunctional β-cells are unable to adjust the quantity of secreted insulin required to maintain normal plasma glucose levels.^[3]^ Therefore, a high level of blood glucose is a sign of DM.^[4]^ Hence, one of the main treatment goals is to maintain stable blood glucose values. The therapy generally comprises oral medications (e.g., biguanides, first-and second-generation sulfonylureas, meglitinides, or sodium-glucose cotransporter-2 inhibitors), non-insulin injections, or insulin injections that are administered to improve insulin deficiency or impaired insulin conditions.^[3]^ Non-pharmacological interventions, such as a modified diet, weight control, regular exercise, physical activity, and bariatric surgery, can also be part of the treatment.^[5]^ Patients who are beginning their therapy require counseling regarding their treatment. After beginning medication, they require regular monitoring. Therefore, it is important to understand the latest and most effective counseling approaches that can be used to treat patients with DM.

Counseling is a professional activity that entails helping clients individually or in groups, or as couples and families, deal with various problems. It is practiced by counselors, psychiatric nurses, psychologists, physicians, pharmacists, and other health care teams. These professionals play a vital role in assisting patients and their families to adjust to chronic illness and assess quality-of-life (QoL) issues related to the disease by educating and advising them regarding adherence to their medication regimens and monitoring plans, thus monitoring their therapy outcomes.^[6]^ Healthcare teams may cooperate in formulating a specific package of information and advice targeting the individual’s care requirements. Such coordinated efforts of health care teams result in improved patient compliance with their medication regimens, more effective drug efficacy monitoring, and the receipt of feedback.^[7]^ Currently, patients with chronic illnesses mainly receive primary care and are trained in self-management. This approach is critical for patients to achieve their personal health goals and communicate effectively with health care practitioners.^[8]^ DM counseling includes monitoring related outcomes such as glycated hemoglobin A (HbA_1c_) levels, daily self-care, and other issues such as blood glucose measurement and compliance with diet, exercise, and medication regimens.^[9]^

Digital approaches, such as teleconsultations, telehealth, or telemedicine, provide solutions for optimizing DM patients’ management of their symptoms and their exacerbation in contexts of restricted contact and inability to access care in person.^[10]^ Patients can communicate and discuss their situations with health workers, who can help them to maintain their self-management regimes using smartphone apps. Their physicians can help them choose suitable types of physical activity and provide guidance on staying physically active by sharing illustrative exercise videos. Physicians or DM educators can also ensure drug compliance, help patients procure their medication, and educate them on how to cope with a hypoglycemic crisis via online consultations. Patients can also have discussions with nutritionists on how to maintain a healthy diet and order the glucose strips online after consulting pharmacists.^[11]^ A study by Röhling et al^[12]^ showed that a telemedicine-based system combined with an approach involving a team of healthcare professionals could be successfully implemented in an inpatient setting and directly improve the quality of care.

Previously published systematic review papers analyzed telemedicine’s effectiveness in managing T2DM, showed a significant reduction in HbA_1c_ and had impacts on secondary outcomes such as mental and physical QoL compared to the control group.^[12–14]^ This evidence motivated us to explore telemedicine counseling strategy in DM management further.^[14]^ We also assessed patients’ adherence as the secondary outcome, which has not been assessed in other reviews. We tried to apply the results to the current counseling technique used in the ongoing coronavirus disease 2019 (COVID-19) pandemic, particularly by professional healthcare in Indonesia.

Telemedicine has emerged as an essential digital platform for implementing this approach, particularly during COVID-19, which has compelled health centers to limit in-person clinical visits to prevent the spread of the virus. The governments of various countries have also launched measures to control the spread of infection, such as lockdowns, quarantine, and social distancing.^[15]^ The likely effects of lockdowns and social distancing on DM patients would have been little physical activity, necessary changes in their dietary habits because of restrictions in food supplies, and difficulties in obtaining their anti-diabetic medication and glucose strips. Moreover, they would have been unable to see their physicians in person to receive regular advice and follow-up care at the clinics. This situation has thus transformed DM management into self-management practices, and the method by which healthcare providers and DM patients communicate has become virtual.^[11]^

We tried to make up the newest studies analysis in the recent 10 years as there was a similar systematic review and meta-analysis with studies ranging from 20 years before. Adding a telemedicine approach to conventional or face-to-face T2DM disease management strategies resulted in a slight decrease in HbA_1c_. We believe that in the face of this pandemic, all healthcare providers are willing to work toward resolving the issues that arise in the service of their patients. This narrative review was conducted to help healthcare professionals provide the best possible health services for DM patients and to consider an optimal counseling approach that could be applied during the ongoing COVID-19 pandemic, which restricts face-to-face meetings. Accordingly, we evaluated the effectiveness of telemedicine as a counseling tool for patients with DM, considering blood glucose levels as the primary outcome.

## 2. Material and Methods

### 2.1. Ethical statements

No ethical approval was necessary for this study because it was a literature-based study. This review was conducted following the Scale for the Assessment of Narrative Review Articles (SANRA).^[16]^

### 2.2. Design

Besides the SANRA guideline,^[16]^ this narrative review was performed and reported according to the Preferred Reporting Items for Systematic Reviews and Meta-Analyses guidelines of 2020.^[17]^ A literature review is described as published materials that provide an examination of recent or current literature. which can cover a wide range of subjects at various levels of completeness and comprehensiveness and may include research findings.^[18]^ According to Sutton et al,^[19]^ a narrative review is defined as a legacy model of a review criticized during the early years of the systematic review movement because of its lack of transparency, and a narrative review serves a continuing role when performed more systematically in orienting research into a broader field.

We did not perform a partial systematic review and meta-analysis owing to time constraints and the limited number of articles available for analysis. We conducted a panel discussion to enable us to complete the quality assessment.

### 2.3. Search strategy and study eligibility

We conducted a literature search by title and abstract in the EBSCO, Embase, PubMed, and Web of Science electronic databases from October to November 2021. In line with the Patients, Intervention, Comparator, and Outcomes search strategy, we applied the following keywords: “diabetes mellitus” AND “telemedicine” AND “counseling” AND “glucose level.”

Randomized controlled trials (RCTs) performed to determine and correlate the effectiveness of telemedicine and conventional counseling for patients with DM were included in the review. We only included full-text articles published in English in the last 10 years. In both telemedicine and traditional in-person contexts, counseling was defined as monitoring treatment and explaining the following aspects to patients: control and measurement of blood glucose levels, information on medication, monitoring of therapy, and health management.

### 2.4. Study selection

Three reviewers and a principal investigator (PI) searched electronic databases. Next, the reviewers screened the titles and abstracts and independently screened the full texts of potentially eligible articles. Disagreements regarding the study design, intervention criteria, and outcome measures were resolved through discussion.

### 2.5. Data extraction and summarized

The 3 reviewers extracted the data independently and checked it by the PI. The reviewers resolved any disagreements through discussion and consultation with the PI. The following data were extracted from the included articles: name(s) of the author(s) and year of publication, location and time of the study, total number of participants in the control and intervention groups, types of telemedicine used, primary and secondary outcome measures, study results, and conclusions.

## 3. Results

### 3.1. Search results

**Figure 1.**
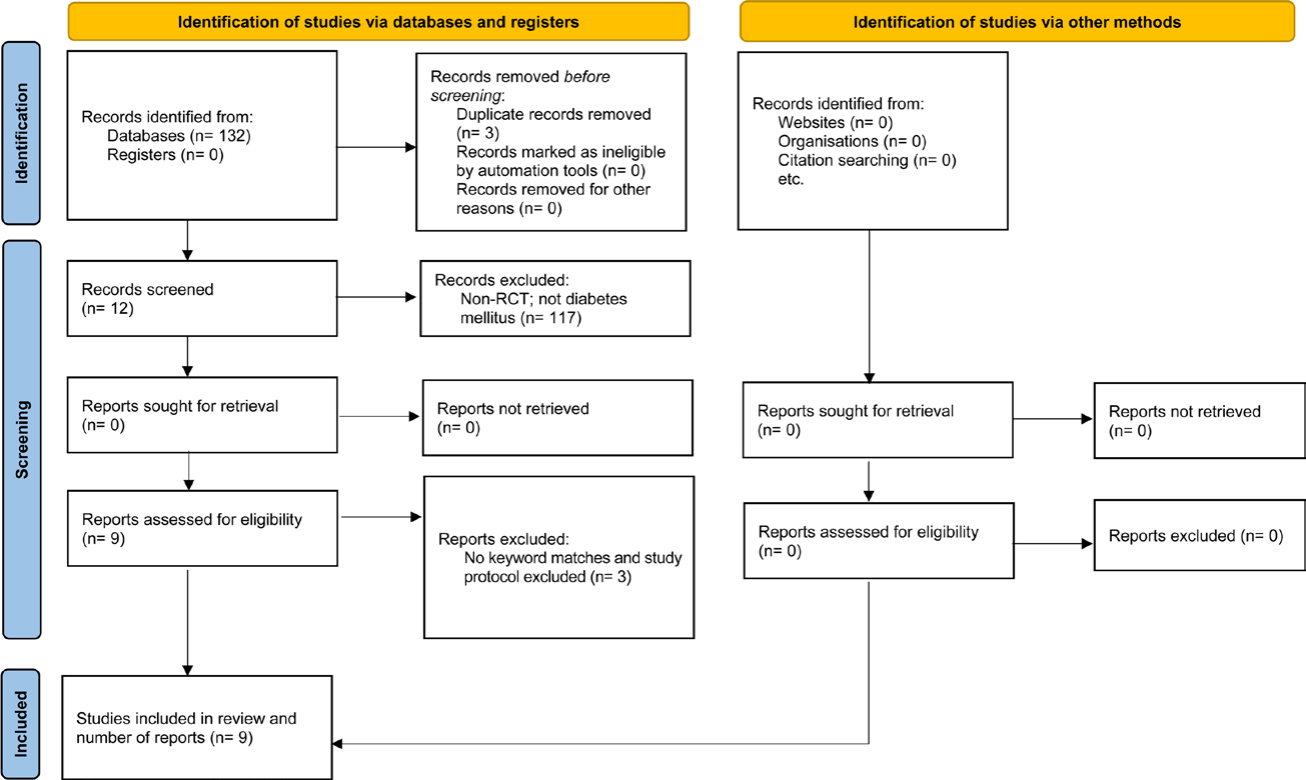
PRISMA flow diagram of included articles. PRISMA = Preferred Reporting Items for Systematic Reviews and Meta-Analyses.

The search of electronic databases yielded 132 articles. After eliminating duplicate articles, 129 remained. After screening the titles and abstracts, 12 articles remained. Finally, 3 studies were excluded because they did not meet the inclusion criteria. In total, we have 9 articles assessed in this review. Figure 1 depicts the process used to select the articles.

### 3.2. Characteristics of the included studies

Table 1 provides a detailed summary of the articles included in the review. The studies in the articles were conducted in South Korea,^[20,27]^ the United States,^[21]^ Australia,^[22]^ Norway,^[23,24]^ Greece,^[25]^ Israel,^[26]^ and Bangladesh.^[28]^ The date range for the studies was 2007 to 2020.

**Table 1:** Table of characteristic study.

No.	Author (years)	Research location and period	Patients age	Type of diabetes	Current treatment received	Type of telemedicine	HbA_1c_ level (%)		
							Base	Final visit	Change
1.	Kim H-S (2007)^[20]^	South Korea (2003–2004)	The mean ages were 47.5 ± 9.1 (control group) and 46.8 ± 8.8 (treatment group)	T2DM	Insulin and oral anti-diabetic medication	● SMS ● Cellular phone or wired Internet ● Website	● Control group: 7.59 ± 1.09 ● Intervention group: 8.09 ± 1.72	● Control group: 7.66 ± 0.91 ● Intervention group: 6.94 ± 1.04	● Control group: +0.07
									● Intervention group: −1.15
2.	Sacco WP et al (2009)^[21]^	USA (September 2001–August 2003)	18–65	T2DM with HbA_1c_ level > 6.5%	Insulin (53% participants) and oral anti-diabetic medication (86% participants)	● Telephone calls	● Control group: 8.5 ± 2.01 ● Intervention group: 8.4 ± 1.37	● Control group: 7.8 ± 1.3 ● Intervention group: 7.4 ± 1.12	● Control group: −0.7 ± 0.71 ● Intervention group: −1 ± 0.25
3.	Marios T et al (2012)^[22]^	Australia (May 2007–October 2010)	18–80	T2DM with FPG > 7.0 mmol·L-1 and HbA_1c_% level > 6.5.	N/A	● Telephone calls	● Control group: 7.53 ± 1.68 ● Intervention group: 7.90 ± 2.39	● Control group: 7.12 ± 0.79 ● Intervention group: 7.61 ± 2.08	● Control group: −0.41 ± 0.89 ● Intervention group: −0.29 ± 0.31
4.	Torbjørnsen A et al (2014)^[23]^	Norwegia (2011–2013)	≥18 yr	T2DM and HbA_1c_ level ≥ 7.1% included (≥54.1 mmol/mol)	● No medication (9 participants) ● Only oral agents (63 participants) ● Only injections agent (19 participants) ● Combination oral and injection (40 participants)	● FTA ● FTA-HC	● Control group: 8.3 ● Intervention group: ± 1.FTA: 8.1 2.FTA-HC: 8.2	● Control group: 8.0 ● Intervention group: ± 1.FTA: 7.8 2.FTA-HC: 7.8	● Control group: −0.39 ● Intervention group: 1.FTA: −0.23 2.FTA-HC: −0.41
5.	Holmen H et al (2014)^[24]^	Norwegia (2011–2013)	≥18 yr	T2DM with HbA_1c_ level ≥ 7.1% (≥54.1 mmol/mol)	● Diet only (9 participants) ● Oral agents only (63 participants) ● Injection only (19 participants) ● Combination of oral and injection (40 participants)	● FTA ● FTA-HC	● Control group: 8.4 ● Intervention group: 1.FTA: 8.1 2.FTA-HC: 8.1	● Control group: 8.2 ● Intervention group: ± 1.FTA: 7.8 2.FTA-HC: 8.0	● Control group: −0.16 ● Intervention group: 1.FTA: −0.31 2.FTA-HC: −0.15
6.	Kotsani K et al (2018)^[25]^	Greece (January–August 2016)	18–39	T1DM for at least 1 year	MDI	● Telephone calls	● Control group: 8.1 ± 1.2 ● Intervention group: 8.3 ± 0.6	● Control group: 7.9 ± 0.8 ● Intervention group: 7.8 ± 1	● Control group: −0.2 ● Intervention group: −0.5
7.	Miremberg H et al (2018)^[26]^	Israel (2016–2017)	Women 18–45 years old	GDM	Insulin injection	● Website	● Control group: 5.2 ± 0.4 ● Intervention group: 5.2 ± 0.33	● Control group: 5.5 ± 1.9 ● Intervention group: 5.3 ± 1.9	● Control group: +0.3 ● Intervention group: +0.1
8.	Sung J-H et al (2019)^[27]^	South Korea (February 2017–August 2017)	The mean age was 33.43	GDM	N/A	● A mobile phone application	● Control group: 33.7 ± 4.3 ● Intervention group: 36.64 ± 5.55	● Control group: 36.75 ± 3.24 ● Intervention group: 35.57 ± 4.69	● Control group: +3.05 ● Intervention group: −1.07
9.	Yasmin F et al (2020)^[28]^	Bangladesh (Baseline: January–December 2014; Endline: January–2015)	The mean age was 53 and 51 years in the intervention and the control group	T2DM	N/A	● Mobile phone-based health reminder system ● 24/7 call center services	● Control group: 8.54 (FBG) ● Intervention group: 8.67 (FBG)	● Control group: 8.13 (FBG) ● Intervention group: 6.8 (FBG)	● Control group: −0.41 (FBG) ● Intervention group: −1.87 (FBG)

FBG = fasting blood glucose, FTA = Few Touch Applications, FTA-HC = Few Touch Applications-Health Counseling, GDM = gestational diabetes mellitus, HbA_1c_ = glycated hemoglobin A, MDI = multiple daily injections of insulin, N/A = not applicable, SMS = short message service, T1DM = type 1 diabetes mellitus, T2DM = type 2 diabetes mellitus.

Nine RCTs were analyzed, encompassing a total of 962 participants. The age range for the intervention and control categories of the participants was 18 to 80 years. The primary outcomes measured and reviewed were blood glucose levels or HbA_1c_, QoL,^[23,24]^ and patient compliance.^[22,25–28]^ The duration of the studies ranged between 3 months^[20,21,25]^ and 1 year.^[24]^

### 3.3. Intervention

The selected studies reviewed the effectiveness of telemedicine as a tool for counseling using conventional counseling as a control. They covered the following topics: pharmacological treatment and therapy, monitoring of therapy outcomes (self-monitoring and joint monitoring with practitioners), monitoring of patients’ treatment adherence, QoL, their activities, and their feedback on their health. The patients’ questions were also considered.

### 3.4. Outcome measures

One of the 9 RCT studies measured HbA_1c_, fasting plasma glucose (FPG), and 2-hour post-meal glucose (2HPMG) levels.^[20]^ Another study measured body mass index (BMI), exercise, diet, DM symptoms, foot care, depressive symptoms, and therapeutic mechanisms but did not measure significant HbA_1c_ levels.^[21]^ In a third study, a home-based exercise program was the primary outcome, and peak oxygen consumption, HbA_1c_ (%), and QoL were secondary outcomes.^[22]^ Using similar methods, 2 studies^[23,24]^ focused on HbA_1c_ levels as the primary outcome, with behavioral changes (physical activity and diet), self-management, and patients’ health-QoL as the secondary outcomes.^[23]^ In addition to symptoms of depression, lifestyle changes (physical activity and dietary habits) were considered in the study.^[24]^ Two other studies used blood glucose levels as the main measurement.^[25,28]^ The first study’s outcomes were morning (fasting), preprandial, and postprandial glucose levels. The outcome measures in the second study were related to the frequency of hospital visits and medication intake adherence combined with a dietary approach, physical exercise, betel nut consumption, tobacco control, and blood glucose control. One study used the actual blood glucose measurement (percentage) as an outcome.^[26]^ Only 1 study measured outcomes related to gestational diabetes: obstetrical outcomes (gestational age at delivery and birth weight), and metabolic outcomes (BMI, weight, percentage of body fat, insulin resistance, and HbA_1c_).^[27]^

### 3.5. Effectiveness of telemedicine counseling

Table 2 summarizes the data extracted from these studies. All the included studies evaluated the effectiveness of telemedicine counseling compared with counseling in the control groups using HbA_1c_ levels. Five of the 9 studies reported that telemedicine counseling was more effective in decreasing HbA_1c_ levels in diabetic individuals than the results of conventional counseling applied to the control group.^[20,25–28]^ However, 1 study did not report HbA_1c_ results.^[21]^ Two studies reported that telemedicine counseling did not significantly improve HbA_1c_ levels, and patients that received usual care had better results.^[22,23]^

**Table 2:** Summary of the included studies.

No.	Author (years)	Interventions	Comparator	Primary outcomes	Secondary outcomes	Results	Conclusions
1.	Kim H-S (2007)^[20]^	Each patient was instructed to input data into the website www.biodang.com and their self-monitored blood glucose levels and drug information, including the kinds and dosages of insulin and oral anti-diabetic medication they used for diabetes control. Each patient also got optimal recommendations back weekly, by an SMS by cellular phone or wired Internet	Participants in the control group met with an endocrinologist once or twice over 12 wk. The endocrinologist gave recommendations on medication, dosage, and lifestyle modifications when they visited the clinic. More individualized and detailed information relating to lifestyle modification is given when the doctor or patient wants to	HbA_1c_, FPG, and 2HPMG levels	N/A	This study shows that HbA_1c_ levels decreased significantly by 1.15% in the intervention group after 12 wk, even though the duration of the study was concise. The intervention group also decreased FPG levels of 0.4 mmol/L, but there was no interaction between group and time. The rate of change in blood glucose levels in the control group was not significant. The average rate of change was +0.07. However, there was no significant difference between the groups	The intervention group showed a significant decrease in HbA_1c_ values, but there is no significant difference from the control group
2.	Sacco WP et al (2009)^[21]^	Patients in the intervention group received telephone coaching with a diabetes coach for 15–20 min. One per week calls for the first 3 mo and 1 bi-weekly call for the remaining 3 mo. Patients also received treatment from an endocrinologist and completed the pretest and post-test	Patients in the control group only received treatment from an endocrinologist and completed the same pretest and post-test measures as the intervention group	HbA_1c_, BMI, diet, exercise, foot care, depressive symptoms, diabetes symptoms, and therapeutic mechanisms	N/A	This study shows significant positive effects on diabetes-related self-efficacy, self-care activities, and self-care goals. The intervention group improved diet, exercise, foot care, depressive and diabetes symptoms, self-efficacy, and awareness of self-care goals. However, this study failed to find a significant effect in A1c and BMI because they were observed only on face-valid self-report measures to reflect a response bias of positive outcomes	The telephone coaching intervention showed a reduction of depression and diabetic symptoms and improved self-efficacy, self-care activities, and self-care goals. However, it failed to show any effect on glycemic control and BMI
3.	Marios T et al (2012)^[22]^	All participants were given a 6-mo individualized walking program. Patients were also asked to complete 180 min per week of exercise, and their venous blood was taken to establish serum levels	Same as the intervention group, the control group were given a 6-mo individualized walking program. They were asked to complete 180 min per week of exercise and their Venous blood was taken to establish serum levels. The differences were that control patients did not receive heart rate monitors and phone calls, but were taught the way to take their pulse to monitor heart rate	A home-based exercise program	Peak VO_2_, HbA_1c_%, and quality of life	Intervention groups patients completed a mean weekly exercise of 138 min while control group patients completed 58 min weekly	This study shows that telemonitoring groups have improved the weekly exercise, the peak VO_2_, treadmill time, and maximum heart rate even though there is no significant HbA_1_c% improvement
						The improvement of peak VO_2_, treadmill time, and maximum heart rate in the intervention group are 5.5% (21.8–23.0), 18% (8.31–9.87), and 3% (101–104). Whereas HbA_1c_% of the telemonitoring group decreased from 7.90 to 7.61, and HbA_1c_% of the control group decreased from 7.53 to 7.12	
		Intervention groups were provided with a heart rate monitor and received weekly phone calls, ranging from 5 to 15 min. Patients were allowed to ask contact their exercise physiologist					
4.	Torbjørnsen A et al (2014)^[23]^	Two intervention groups: 1. Participants used FTA 2. Participants used FTA-HC with a diabetes specialist	Participants received usual care	HbA_1c_ target in Norway is ≤7.0%	Self-management (Health Education Impact Questionnaire, heiQ), behavioral change (these were diet and physical activity), also health-related quality of life (SF-36) questionnaire	The FTA intervention may have taken too long, also even more for the FTA-HC group. There was also a limitation of a more significant opportunity for the participants to influence the results. The mean decrease change of HbA_1c_ in the FTA group is 0.23, while the mean change of HbA_1c_ in the FTA-HC group is 0.41, and for the control group who received usual care is 0.39. The blood glucose measurement remains high. This does not decrease rapidly for both FTA and FTA-HC	FTA interventions with or without HC may not be effective enough. FTA and health counseling did not help lower HbA_1c_ levels compared to controls
5.	Holmen H et al (2014)^[24]^	Two intervention groups: 1. Participants used FTA 2. Participants used FTA-HC by a diabetes specialist	Participants received usual care	HbA_1c_ level	Self-management (heiQ), health-related quality of life (SF-36), depressive symptoms (CES-D), also lifestyle changes (dietary habits, also physical activity)	HbA_1c_ levels decreased in all groups, but there was no significant difference in changes after 1 year. However; in these 3 groups, the mean HbA_1c_ level did not increase to the baseline level It was shown that there was a significant change in self-management in the FTA-HC group, as they alleviated symptoms and improved their ability to manage their health effectively, also better skills in using technical aids	Although there is no significant HbA_1c_ level difference, The FTA-HC with diabetes specialists were indicated to be more effective in their health-self management. and reducing patients’ symptoms
6.	Kotsani K et al (2018)^[25]^	Patients in the intervention group were asked to write down their glucose values ​​in a diary and submit them in 3 ways: via USB connected to a glucose meter, email, or phone call. This group is contacted by telephone every Thursday (10–12 am) for 5–15 min by the nurse-coordinator, discussing possible problems in their disease management and some recommendations based on the data they input	Patients in the control group were asked to write down their glucose values ​​in a diary. The researchers advised patients to submit their data either via USB connected to a glucose meter or by email or collect them to be reviewed at the end of the study. No interaction over the telephone is done in the control group	The measurement of the morning (fasting), preprandial, postprandial glucose level, HbA_1c_	NA	For the intervention group the morning mean values of blood glucose are 120.01 mg/dL (month 1), 105.96 mg/dL (month 2), and 93.18 mg/dL (month 3); the preprandial mean values of blood glucose are 148.23 mg/dL (month 1), 125.59 mg/dL (month 2), and 114.76 mg/dL (month 3); the post prandial mean values of blood glucose are 248.3 mg/dL (month 1), 215.8 mg/dL (month 2), and 193.35 mg/dL (month 3). And for the control group the morning mean values of blood glucose are 107.18 mg/dL (month 1), 105.96 mg/dL (month 2), and 105.17 mg/dL (month 3); the preprandial mean values of blood glucose are 120.66 mg/dL (month 1), 119.91 mg/dL (month 2), 114.76 mg/dL (month 3); the post prandial mean values of blood glucose are 220.92 mg/dL (month 1), 213.22 mg/dL (month 2), and 207.84 mg/dL (month 3)	There has been a significant improvement in the glucose level in the intervention group
7.	Miremberg H et al (2018)^[26]^	Patients reported their glucose level using the web-based app (https://www.glucosebuddy.com) and received individual feedback regarding their daily glycemic control every evening (including weekends). They are expected to ask questions by the app, receive answers immediately related to GDM management aspects	Patients in the control group had their first visit, biweekly visits until 35 wk gestation, and weekly at 35 wk gestation until delivery. Patients were also asked to monitor their glucose level 4 times daily (once during the morning fast and after each main meal), and manually record it on a paper diary to be reviewed at each visit	Patient compliance (expressed as a percentage) is defined as the actual blood glucose measurements/instructed measurements times 100	Diabetes-control parameters: mean blood glucose (mean ± all measured values SD), insulin need treatment, and percentage of off-target measurements (thresholds: fasting > 95 mg/dL and 1-h postprandial > 140 mg/dL)	The patient compliance was higher in the smartphone group compared to the control group (84 ± 0.16% vs 66 ± 0.28%, *P* < .001). The mean blood glucose shows that it was significantly lower in the smartphone group (105.1 ± 8.6 mg/dL vs 112.6 ± 7.4 mg/dL, *P* < .001). Insulin treatment’s overall rate was also more diminished in the smartphone group than the control group (13.3% vs 30.0%, *P* = .044), same with the rates of off-target measurements both fasting (4.7 ± 0.4% vs 8.4 ± 0.6%, *P* < .001) and 1 h postprandial (7.7 ± 0.8% vs 14.3 ± 0.8%, *P* < .001)	Patients in the intervention groups show better outcomes (higher compliance, lower mean blood glucose, lower insulin treatment needs, and lower rates of off-target measurements)
8.	Sung J-H et al (2019)^[27]^	The MM group received standard antenatal care and was given monitoring system devices. Patients recorded their blood glucose concentration and diet using a mobile phone application (Huraypositive Inc, Seoul, Korea). The mobile application received regular messages twice a week about recommendations for adequate diet and exercise during the study period	The CM group received standard antenatal care from obstetricians and endocrinologists, consisting of biweekly visits up to 36 wk of gestation then weekly visits until delivery. Patients were also asked to record their blood glucose concentration 4 times daily and record their intake at each meal	The outcomes divided into obstetrical outcomes (GA at delivery, birth weight, cesarean section) and metabolic outcome (BMI, weight, percentage of body fat, IR, and HbA_1c_) after delivery measured at 4–12 wk postpartum	N/A	Patients that used mobile phone application for the management of GDM had lower BMI (20.22 kg/m^2^ vs 23.72 kg/m^2^), weight (54.31 kg vs 62.58 kg), percentage of body fat (38/12% vs 29.20%), IR (1.97 vs 1.46), and HbA_1c_ (36.75% vs 35.57%) after delivery compared with a conventional management group. No significant difference in glycemic control during pregnancy and perinatal outcome between the MM and CM groups. The mean value of fasting glucose measured in the CM group at 4–12 wk postpartum is 103.63 mg/dL, while in the MM group is 101.43 mg/dL	Patients in MM (intervention) groups show lower levels of the outcomes
9.	Yasmin F et al (2020)^[28]^	Patients received a mobile phone-based health project (reminder system through interactive voice calls and 24/7 call center services). At the hospital, patients were also given recommendations on medication, diet, physical activity, hospital visits, and lifestyle modification measures	Patients in the control group were only getting regular hospital services	Adherence to medication intake and hospital visit practice, dietary practice, physical exercise, betel nut, tobacco control, blood glucose control consists of the fasting blood glucose level and the blood glucose level by 2 h after breakfast. They are also taking advantage of 24/7 call center services	N/A	It showed a positive impact of adherence to medication intake and hospital visits even though some patients abandoned the medication and hospital visit. The intervention group adhered to carbohydrate and total kilocalorie, vegetable, and fruit intake. There were improvements in the mean exercise number per week (from 4.6 to 6.13 d) in the intervention group and reduction of smoke tobacco (from 2.5% to 1.4%) and betel nut use (from 24% to 8%). Mean fasting and mean blood glucose levels 2 h after breakfast decreased from 8.67 to 6.80 and 12.33 to 9.84 in intervention patients. The call center received 5 calls on average during 1 year period from the intervention group	Participants in the intervention group showed improvement in adherence to diet, physical exercise, betel nut, tobacco control, and blood glucose levels control. It showed good medication adherence also hospital visits, but only a few patients used the free 24/7 call center services

2HPMG = 2-hour post-meal glucose, BMI = body mass index, CM = conventional management, FPG = fasting plasma glucose, FTA = Few Touch Applications, FTA-HC = Few Touch Applications-Health Counseling, GA = gestational age, HbA_1c_ = glycated hemoglobin A, MM = mobile management.

Furthermore, although HbA_1c_ levels did not decrease significantly, 1 study reported that the results of telemedicine with health counseling offered by a DM specialist were more significant concerning patients’ treatment, medication adherence, and QoL.^[24]^ Six of the 9 studies also assessed other parameters related to patients’ compliance and QoL. They all reported that telemedicine increased these parameters in patients with DM.

## 4. Discussion

The supplementary data depicts the result of SANRA guideline^[16]^ assessment, http://links.lww.com/MD/I42. All 9 studies assessed have SANRA scores equal to or above 10. The study by Torbjørnsen et al,^[23]^ Holmen et al,^[24]^ and Sung et al^[27]^ had the highest SANRA score of 12. Nine studies were analyzed for the intervention of telemedicine counseling applied in each study related to DM management.

This review compared current evidence from recent studies on the effects of telemedicine counseling and conventional (face-to-face) counseling in patients with DM using glucose levels as the outcome measure. To the best of our knowledge, a review of RCTs comparing telemedicine counseling and conventional (face-to-face) counseling in patients with DM using glucose levels as the outcome measure has not been performed. Thus, the objective of this review was to evaluate the effectiveness of counseling methods in patients with DM. This finding showed that counseling and monitoring patients with DM via telemedicine were more effective than conventional counseling.

Five of the 9 studies reported positive results regarding the effectiveness of telemedicine. For instance, in a study by Kim,^[20]^ the FPG value decreased. In contrast, the HbA_1C_ and 2HPMG values decreased significantly for patients in the intervention group who received counseling on DM treatment and monitoring. In contrast, the values for all outcomes in the control group increased, rather than decreased, after 12 weeks. Patients in the intervention group contacted nurses more frequently than those in the control group received advice based on the latest data, and they confirmed that their present condition could explain this finding. Consequently, they might have been more motivated to control their glucose levels. However, despite the decrease in FPG and 2HPMG levels in the intervention group, most patients did not reach the target FPG and 2HPMG levels. This result may be attributed to the short study duration. Nonetheless, this study found that telemedicine was effective.

Kotsani et al^[25]^ also found that the mean glucose level (morning, preprandial, and postprandial blood glucose) and the mean HbA_1c_ level of the experimental group was significantly reduced compared with those in the nonintervention group. After 3 months of study, the values for all outcomes showed good progress in the intervention group. The HbA_1c_ level decreased by 0.5% in the intervention group compared with the 0.2% decrease in the control group.

Miremberg et al^[26]^ found that patients in the intervention group had better compliance values than those in the control group. This result affected other outcome parameters such as mean blood glucose level, insulin treatment rate, off-target insulin rate, and the 1-hour postprandial blood glucose level. Consequently, the values of these parameters were improved. The patients’ compliance values were calculated as the actual blood glucose measurements/instructed measurements multiplied by 100.

Sung et al,^[27]^ who studied gestational DM patients, also found that patients in the intervention group had better post-delivery metabolic outcomes than those in the control group. This result may be attributed to their receipt of regular, twice-weekly messages, communication with healthcare providers, and adherence to dietary and exercise recommendations. The control group communicated only with the healthcare providers when they visited the clinic.

Yasmin et al^[28]^ also found that mobile healthcare positively affected the intervention group. This group obtained better results for blood glucose levels 2 hours after breakfast and fasting blood glucose than the control group. These positive results could be because the intervention group received interactive voice calls every Friday, every 10 days, and on national holidays. Patients could also access a physician and obtain counseling by contacting 24/7 call center services. These call-based interactions supported them through recommendations related to hospital visits, medications, physical exercise, diet, and other lifestyle modifications. The patients also received reminders notifying them when their hospital visits were scheduled. These interactions positively affected medication adherence and hospital visits, although some patients abandoned their medication and hospital visits because of financial constraints.

However, 3 studies reported that telemedicine did not significantly improve HbA_1c_ levels. The study by Marios et al^[22]^ showed that patients who were instructed to exercise for 180 minutes per week and were given counseling telephonically did not demonstrate significant decreases in blood glucose levels, even though the mean exercise duration for the intervention group was 138 minutes. By contrast, the control group showed better results since more than 138 minutes of exercise per week or more than 1392 (kcal) of energy expended through weekly exercise are required to achieve improved glycemic control. The findings of this study are consistent with the guidelines of the American Heart Association, which state that a minimum weekly exercise routine amounting to 150 minutes of moderately intense or 90 minutes of high-intensity activity is required to improve glycemic control. However, examination of other outcomes showed that the intervention group had better results for maximum oxygen consumption, exercise (treadmill) test time, and maximum heart rate.

In addition, Torbjørnsen et al^[23]^ indicate that telemedicine may not be sufficiently effective in decreasing HbA_1c_ levels. In this study, there were 2 intervention groups: in 1 group, the Few Touch Application (FTA) was applied, and in the other, FTA with health counseling (FTA-HC) was applied. In both groups, the blood glucose levels remained high and did not show a rapid decrease. FTA intervention may have been too time-consuming, especially for the FTA-HC group. The availability of new software could have enhanced the ability of participants to influence the results, thereby changing the intervention. The use of apps and health counseling in the experimental group did not reduce HbA_1c_ levels as the primary outcome relative to this outcome in the nonintervention group. However, utilization of the app improved the participants’ skills in navigating self-management of health care and symptom relief, with or without additional health counseling.

Holmen et al^[24]^ found that HbA_1c_ levels were reduced in all intervention groups under similar conditions. However, the changes observed in the different groups after 1 year did not differ significantly. Nevertheless, for secondary outcomes, there were significant changes in self-management and lifestyle in the FTA-HC intervention group. Individuals in this group demonstrated enhanced skills related to technical aids and were sufficiently able to reduce symptoms and manage their health effectively. This improvement is an essential requirement for the daily self-management of DM. It is also reasonable to assume that if self-administered interventions improve HbA_1c_ levels, they must entail healthy eating, adequate physical activity, and medication adherence. Therefore, it can be concluded that the FTA-HC intervention, which included health counseling with DM specialists, led to more improvements in self-management and lifestyle changes compared to the FTA group, whose members did not undergo health counseling. Thus, face-to-face counseling may contribute to better health and quality of life in patients with DM.

Although the method used by Sacco et al^[21]^ was almost the same as that used in other studies, their results differed. They did not find a significant effect on HbA_1c_ levels or BMI. In this study, the intervention group had weekly telephonic coaching sessions with a DM coach, each lasting 15 to 20 minutes for the first 3 months, and subsequently had fortnightly sessions during the last 3 months. The reason for not measuring HbA_1C_ levels and BMI significantly was that significant effects were observed only on valid face self-report measures. Positive outcomes could reflect a response bias. In addition, it may be unreasonable to expect a high level of reduction in HbA_1c_ and BMI, given the brevity of the sessions and the semi-structured nature of coaching calls. However, this study showed significant positive effects on patients’ diabetes-related self-efficacy, adherence to self-care activities (e.g., diet, physical exercise, and foot care), and awareness of self-care goals. They also showed decreased depressive and DM symptoms.

Most studies found that telemedicine counseling improved the quality of counseling methods for DM patients and led to decreased blood glucose levels, higher medication and treatment adherence levels, and a better QoL among patients. One study found that telemedicine and health counseling by DM specialists led to improved QoL and self-care management among recipients.^[24]^

In this study, we focused only on DM management. However, telemedicine counseling positively affected the outcomes of other diseases and hypertension management. A review by Hoffer-Hawlik et al^[29]^ demonstrated that technologies imparted through telemedicine for hypertension management significantly affected blood pressure outcomes in most 14 low-and middle-income countries studies. These findings are consistent with previous reviews on telemedicine interventions for hypertension control in high-income countries. For example, an overall summary was carried out by Wang et al,^[30]^ estimated that there was an improvement in clinical blood pressure control.

Although telemedicine has proven advantageous in disease management, it also has a few disadvantages, such as the absence of a physical examination by healthcare experts. The weakness of telemedicine causes difficulties in diagnosis in a virtual clinic. Furthermore, most patients are older individuals with limited technological skills, some of whom do not know how to access telemedicine or may even be unaware of this treatment option.^[31]^

## 5. Conclusion

Most of the studies included in the review found that counseling applied in a telemedicine context was more effective than the conventional counseling method. A critical review finding was decreased blood glucose levels, increased treatment and medication adherence, and improved QoL among patients with DM. In light of the findings of 1 of these studies, a combination of telemedicine and conventional counseling with more health counseling provided by DM specialists could be considered. This approach will increase patient knowledge and enable healthcare providers to provide routine supervision. Data analysis is facilitated by using specific apps and electronic health recordings. Finally, patients can be monitored at home without visiting the clinic, avoiding face-to-face contact, following the “new normal” in the COVID-19 pandemic context.

### 5.1. Strengths and limitations

Although the narrative review yielded 9 studies, and several limitations are associated with the methodology used. First, a simple panel discussion was conducted among the authors to assess the quality of the articles, and the risk of bias was not considered. These conditions may have undermined the validity of the findings and led to errors. Second, the search for existing studies may not have been sufficiently comprehensive; we searched only 3 databases due to time limitations so some articles might have been missed. Third, some articles or references may have been missed because of Medical Subject Headings terms or keywords. We also limited our search to include only studies conducted in English; therefore, this might have led to missing relevant studies in other languages. We conducted a narrative review because we wrote this paper after winning the Center of Excellence in Higher Education for Pharmaceutical Care Innovation competition, Universitas Padjadjaran, Bandung, Indonesia, and were restricted by time constraints. More research is needed to investigate further the points and themes raised in this study.

Consequently, we conducted a meta-analysis as a follow-up study. However, we attempted to address these gaps by following the latest Preferred Reporting Items for Systematic Reviews and Meta-Analyses guidelines when conducting the literature search. The strength of this study relates to the adherence of DM patients to the current RCT studies.

### 5.2. Clinical implications

The findings of this review indicate that telemedicine counseling may be effective for patients with diseases, such as DM, that require long-term therapies for their management. They suggest that implementing a combined (hybrid) counseling method, entailing a mix of conventional (face-to-face) and telemedicine counseling in countries such as Indonesia, is appropriate and efficient in the current context of the COVID-19 pandemic.

## Acknowledgments

We appreciate the support provided by the 1st International Students Conference (ISC) committee, which granted permission to publish the findings of this narrative review.

## Author contributions

All authors made substantial contributions to the conception and design, acquisition of data, or analysis and interpretation of data; took part in drafting the article or revising it critically for important intellectual content; agreed to submit to the current journal; gave final approval of the version to be published; and agreed to be accountable for all aspects of the work. The authors contributed to the work reported in detail below:

**Conceptualization:** Clara Fernanda Kusuma, Levina Aristawidya, Chairani Putri Susanti.

**Data curation:** Clara Fernanda Kusuma, Levina Aristawidya, Chairani Putri Susanti.

**Formal analysis:** Angga Prawira Kautsar.

**Funding acquisition:** Angga Prawira Kautsar.

**Investigation:** Clara Fernanda Kusuma.

**Methodology:** Angga Prawira Kautsar.

**Project administration:** Angga Prawira Kautsar.

**Resources:** Angga Prawira Kautsar.

**Software:** Angga Prawira Kautsar.

**Supervision:** Angga Prawira Kautsar.

**Validation:** Chairani Putri Susanti.

**Visualization:** Levina Aristawidya.

**Writing – original draft:** Clara Fernanda Kusuma, Levina Aristawidya, Chairani Putri Susanti.

**Writing – review and editing:** Angga Prawira Kautsar.

## Supplementary Material


